# Cost effectiveness and value of information analyses of islet cell transplantation in the management of ‘unstable’ type 1 diabetes mellitus

**DOI:** 10.1186/s12902-016-0097-7

**Published:** 2016-04-09

**Authors:** Klemens Wallner, A. M. James Shapiro, Peter A. Senior, Christopher McCabe

**Affiliations:** Department of Emergency Medicine, University of Alberta, 736 University Terrace Building, 8303 - 112 Street, Edmonton, AB T6G 2T4 Canada; Clinical Islet Transplant Program, Alberta Diabetes Institute, University of Alberta, 2000 College Plaza, 8215 - 112 Street, Edmonton, AB T6G 2C8 Canada; Department of Surgery, University of Alberta, Edmonton, AB Canada; Division of Endocrinology and Metabolism, Department of Medicine, University of Alberta, Edmonton, Canada

**Keywords:** Type 1 diabetes, Islet transplantation, Beta cells, Cost-effectiveness analysis, Value of information, Intensive insulin therapy, Edmonton protocol, Markov model, Scenario analysis

## Abstract

**Background:**

Islet cell transplantation is a method to stabilize type 1 diabetes patients with hypoglycemia unawareness and unstable blood glucose levels by reducing insulin dependency and protecting against severe hypoglycemia through restoring endogenous insulin secretion. This study analyses the current cost-effectiveness of this technology and estimates the value of further research to reduce uncertainty around cost-effectiveness.

**Methods:**

We performed a cost-utility analysis using a Markov cohort model with a mean patient age of 49 to simulate costs and health outcomes over a life-time horizon. Our analysis used intensive insulin therapy (IIT) as comparator and took the provincial healthcare provider perspective. Cost and effectiveness data for up to four transplantations per patient came from the University of Alberta hospital.

Costs are expressed in 2012 Canadian dollars and effectiveness in quality-adjusted life-years (QALYs) and life years. To characterize the uncertainty around expected outcomes, we carried out a probabilistic sensitivity analysis within the Bayesian decision-analytic framework. We performed a value-of-information analysis to identify priority areas for future research under various scenarios. We applied a structural sensitivity analysis to assess the dependence of outcomes on model characteristics.

**Results:**

Compared to IIT, islet cell transplantation using non-generic (generic) immunosuppression had additional costs of $150,006 ($112,023) per additional QALY, an average gain of 3.3 life years, and a probability of being cost-effective of 0.5 % (28.3 %) at a willingness-to-pay threshold of $100,000 per QALY. At this threshold the non-generic technology has an expected value of perfect information (EVPI) of $260,744 for Alberta. This increases substantially in cost-reduction scenarios. The research areas with the highest partial EVPI are costs, followed by natural history, and effectiveness and safety.

**Conclusions:**

Current transplantation technology provides substantial improvements in health outcomes over conventional therapy for highly selected patients with ‘unstable’ type 1 diabetes. However, it is much more costly and so is not cost-effective. The value of further research into the cost-effectiveness is dependent upon treatment costs. Further, we suggest the value of information should not only be derived from current data alone when knowing that this data will most likely change in the future.

**Electronic supplementary material:**

The online version of this article (doi:10.1186/s12902-016-0097-7) contains supplementary material, which is available to authorized users.

## Background

Islet cell transplantation is a method for stabilizing certain type 1 diabetes (T1DM) patients with hypoglycemia unawareness and unstable blood glucose levels [1, 2]. For these patients conventional management with intensive insulin therapy can be life-threatening [3]. In allogeneic islet cell transplantation the islets, isolated from deceased donors, containing glucose-responsive insulin-producing beta cells, are transplanted to replace the patient’s own beta cells, previously destroyed by the chronic autoimmune disease T1DM [3–5]. This treatment has substantially improved the lives of patients, by reducing glycemic lability, risk of severe hypoglycemia, so-called “death-in-bed events”, long-term cognitive and physical disability, and in many cases has led to decrease or discontinuation of exogenous insulin therapy for variable periods of time [6–8].

In July 2000 the Edmonton protocol for islet cell transplantation, developed at the University of Alberta Hospital (Edmonton, Canada), reported promising results of achieving sustained insulin independence in seven consecutive patients [4]. However, five-year insulin independence rates were only about 10 % [9]. Substantial refinements of transplant procedure and immunosuppression regimen, have led to insulin independence rates of 60 % at four years and 50 % at five years, rates that now match those results obtained by whole pancreas transplantation [2, 8, 10, 11]. Further, graft survival, the main factor in protection against severe hypoglycemia, is 82 % at five years [8, 9]. Of the 166 patients that had islet cell transplantations at the University of Alberta Hospital between 1999 and July 2012, 79 % still had graft survival in 2012 and therefore protection from severe hypoglycemia [2]. Graft survival was defined here, in agreement with Barton and colleagues, as having either full graft function (independence from exogenous insulin for ≥14 consecutive days) or partial graft function (no insulin independence, but with C-peptide secretion ≥0.3 ng/mL) [8].

Between 120,000 and 300,000 Canadians have T1DM [12–14]. Of these about 10 % are especially sensitive to insulin and have defective counter-regulatory hormonal responses, putting them at higher risk of neuroglycopenia due to severe hypoglycemia [15]. Additionally, up to 10 % of T1DM mortality is due to hypoglycemia [16, 17]. Consequently, between 15,000 and 30,000 Canadian patients could potentially benefit from islet cell transplantation. However, patients must meet extensive eligibility criteria, including absence of: significant renal impairment, active infection (tuberculosis, HIV), desire for fertility, malignancy, thrombophilia or coagulopathy, substance abuse, insulin requirements of > 1.0 u/kg/day, and severe or untreated cardiac disease [2]. Additionally, there are many barriers to the widespread adoption of islet cell transplantation, including limited donor availability and the risks of post transplant immunosuppression [18].

The purpose of our study was to evaluate the cost-effectiveness of allogeneic islet cell transplantation, as provided at the University of Alberta Hospital. We compared the costs and benefits of allogeneic islet cell transplantation and intensive insulin therapy in transplant eligible patients. Our study was conducted from the perspective of the provincial health care payer (Alberta Health Services), using the patterns of care and costs observed at the University of Alberta Hospital. In addition, we examined the value of further research to reduce current uncertainties in the evidence to inform future reimbursement decisions.

## Methods

Our analysis was performed within a Bayesian decision-analytic framework using stochastic modelling techniques. We used a discrete state-transition Markov model to simulate marginal differences in clinical effects and costs, resulting from the two treatment alternatives. The population was a hypothetical cohort of patients who met the transplantation inclusion criteria and have the below defined characteristics. We followed current best practice modeling guidelines [19–21]. Our methods for characterising the value of further research were built upon those previously described by Hall and colleagues [22].

### Treatments compared

Intensive insulin therapy (IIT) is variable but involves a form of frequent self-monitoring of blood glucose and three or more daily insulin injections, or equivalent therapy with an insulin pump. IIT generally includes the use of a long acting basal insulin (e.g., glargine or detemir) and multiple doses of short acting insulin (e.g., glulisine, aspart, lispro), with highly variable dosage, usually 0.6 u/kg/day. In our model, we simulated mean results of either best manual or device-based injection and monitoring, plus adherence to behavioural recommendations for hypoglycemia unawareness.

In contrast to IIT, islet cell transplantation is performed via injection into the liver with on average 5783 islet equivalents per kg for each procedure [2, 9]. Immunosuppression is necessary for as long as there is graft survival. External insulin injections are only needed when graft survival decreases and partial insulin dependence recurs. The medication used as of 2012 is summarised in Table 1. Generic and non-generic medication was assumed to be equally effective. We excluded other treatments involving pancreas and/or kidney transplantation as comparators because their medical risk-benefit ratio was considered to be too high for patients with our baseline characteristics, largely due to the risks associated with major surgery [8, 18].

**Table 1:** Cost parameters (in 2012 Canadian dollar per patient)

Source of costs	Mean	SD	RSD	Distribution	Hyperparameters	Source^a^
Pre-transplant visit	569					University of Alberta Hospital
Transplantation (including initial medication, based on 4 day stay: 1 pre-op and 3 post-op)	91,414		15.0 %	Log-Normal	**μ = 11.412029**	University of Alberta Hospital
					**σ = 0.149166**	
Total costs per transplantation (including all costs in the 23 days after a transplantation)	94,765			(via input)		
*Medication and follow-up (from day 4 post-op onward)*						
Tacrolimus (per month)	450		7.5 %	Log-Normal	**μ = 6.106443**	University of Alberta Hospital
					**σ = 0.074895**	
Mycophenolate mofetil (MMF; per month)	500		7.5 %	Log-Normal	**μ = 6.211803**	University of Alberta Hospital
					**σ = 0.074895**	
Alemtuzumab (once per transplant)^b^	0					University of Alberta Hospital
Basiliximab (once at 2nd transplant for about half of patients instead of Alemtuzumab)	3000					University of Alberta Hospital
Valganciclovir (for 14 weeks)	5000	375	7.5 %	Log-Normal	**μ = 8.514389**	University of Alberta Hospital
					**σ = 0.074895**	
Anakinra (total for remaining 3 days after discharge)	574	43	7.5 %	Log-Normal	**μ = 6.349825**	University of Alberta Hospital
					**σ = 0.074895**	
Immunosuppression (per cycle, drugs: see above)	713	53	7.5 %	Log-Normal	**μ = 6.56667**	Calculated via data from University of Alberta Hospital
					**σ = 0.074895**	
Generic immunosuppression (per cycle)	238	18	7.5 %	Log-Normal	**μ =5.46808**	Calculated as 1/3 of cost above (price reduction for generic version based on market prices)
					**σ = 0.074895**	
Costs for insulin therapy in graft survival state (per cycle)	64			(via input)		Calculated via data from [33] and based on [10] as 40 % of costs with intensive insulin therapy
Post-transplant check-up visit (at week 1, 2, at 6 months and 1 year and then once a year)	556	42	7.5 %	Log-Normal	**μ = 6.31800**	University of Alberta Hospital
					**σ = 0.074895**	
*Total per cycle (per year) costs for immunosuppression, follow-up and insulin (if applicable)*						
Full graft function for the first 6 months	1886 (30,175)			(via inputs)		Calculated via data from University of Alberta Hospital
Full graft function after the first 6 months	747 (11,956)			(via inputs)		Calculated via data from University of Alberta Hospital
Partial graft function for the first 6 months	1950 (31,196)			(via inputs)		Calculated via University of Alberta Hospital data and [33]
Partial graft function after the first 6 months	811 (12,977)			(via inputs)		Calculated via University of Alberta Hospital data and [33]
Intensive insulin therapy						
*Average healthcare costs of treating people with newly diagnosed diabetes (first 10 years) per cycle (per year)*						
Without complications for type 1 and 2 diabetes	159 (2552)	12	7.5 %	Log-Normal	**μ = 5.06920**	Corrected data from [33]
					**σ = 0.074895**	
With complications for type 1 and 2 diabetes	602 (9632)	120	20.0 %	Gamma	**α = 25.00000**	Calculated from corrected data in [33]
					**β = 24.0796**	
Other costs (per occurrence)						
Average extra costs of initial immunosuppressive or other complications	600	180	30.0 %	Gamma	**α = 11.1111**	Assumption based on [2] and expert opinion
					**β = 54.0000**	
Average extra costs of major immunosuppressive complications	6500	1300	20.0 %	Gamma	**α = 25.0000**	Assumption based on [2] and expert opinion
					**β = 260.0000**	

Bold values were directly used as model inputs and for calculating mean, standard deviation (SD) and relative standard deviation (RSD; i.e., SD as percentage of the mean). Other values are rounded and were incorporated into bold values. Superscripts: ^a^ Source relates to mean values. SD values were the authors’ estimations due to lack of data. ^b^ At the time of our study it was provided at no charge through a compassionate release program

### Baseline patient characteristics

Based on findings in two studies we defined the following baseline characteristics [2, 23]. Additional to fulfilling the eligibility criteria above, we assumed 55 % of our cohort to be female, on average 47 years old, with a diabetes duration of 29.4 (standard deviation = 11.3) years, a weight of 71.1 (11.8) kg, a BMI of 24.8 (3.1), a glycated haemoglobin level of 8.2 % (1.3 %), insulin requirements of 0.6 (0.17) u/kg/day, and only minor to moderate comorbidities [2].

### Perspective

The analyses are presented from the perspective of the health care payer. Therefore, we considered only health effects for the patients, and only direct costs faced by the healthcare provider for those patients. Effectiveness is expressed in quality-adjusted life-years (QALYs) in order to measure the impact of therapy on both quality of life (QOL) and life expectancy. Secondary analyses using life years are also reported. All costs are measured in 2012 Canadian dollars, with necessary adjustments made using the Canadian consumer price index for health and personal care [24]. Not included in this analysis are costs falling on the patient’s family and the impact of health problems on economic activity (productivity costs).

### Model structure

Cycle length was one-sixteenth of a year (~23 days) with a time horizon of 1000 cycles (62.5 years). Cycle length was chosen following consultation with clinical experts, to allow for the appropriate characterisation of the clinical follow-up of transplant patients, including repeat transplantations. Although some patients have received additional transplantations following extremely late graft failure [25], these cases are very rare and so are not included in the model structure. All patients entered the model at the same time and our time horizon covered their remaining lifetime.

#### Transplantation arm

**Fig. 1:**
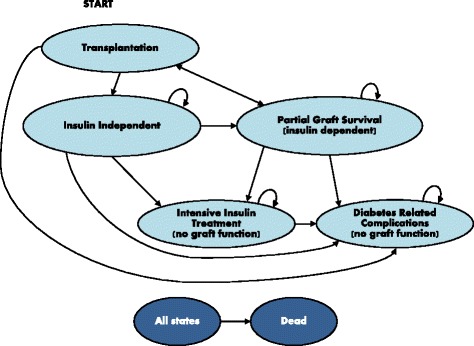
Summary model structure (simplified). The transplantation arm includes all six states. In contrast, the comparator arm only includes the Intensive Insulin Treatment states with and without diabetes-related complications and the Dead state

The transplantation arm had six states (Fig. 1): transplantation, insulin independent with full graft survival, partial graft survival and insulin dependent, IIT without complications, IIT with diabetes-related complications and dead. Each patient could have up to four transplantations. All patients started in Transplantation from where they could move to Insulin independent, Partial graft survival, IIT with diabetes related complications and – as from all states – to Dead. Patients could not move directly from Transplantation to IIT without complications. Due to the strict eligibility criteria all transplant patients were assumed to have at least some initial graft survival unless they experienced diabetes-related complications.

Patients in the Insulin independent state could remain in this state or move to Partial graft survival and to one of the IIT states (with or without complications). The Insulin independence state could be entered from the transplantation state only.

Patients could enter Partial graft survival only from the Transplantation or Insulin independent states. Re-transplantation was an option only for patients in Partial graft survival. In the first six months patients in partial graft survival state were either re-transplanted or moved to IIT with or without complications. After the first six months they could now either remain in Partial graft survival or move to IIT with or without complications. Patients in IIT without complications could stay there or move to IIT with complications. Patients in IIT with complications could only remain there or move to Dead.

#### The comparator arm

The comparator arm had the IIT states with and without complications, and Dead. These were the same states as in Fig. 1 but without the Transplantation, Insulin independent and Partial graft survival states. The costs, utilities and transition probabilities for these states were presumed to be the same as for the corresponding states in the transplantation arm.

#### Modelling of complications

Our model included three groups of complications: 1) initial transplant or immunosuppressive-related complications within the Transplant state, 2) subsequent major immunosuppressive-related complications within the graft survival states, and 3) diabetes-related complications without graft function as a separate state. We modelled the first two complications as being a weighted part of the relevant state costs and utilities. This was similar to our approach for the complications state where the model structure has been simplified by aggregating serious diabetes complications, additional to the baseline characteristics, into a single state. Modelling all of the different complications and combinations of those complications would have made the model substantially more complex, likely requiring some form of patient level simulation. Given the primary health benefit of islet cell transplantation is the reduction in the risk of hypoglycemia associated mortality, this aggregation of other diabetes related complications was judged to be an acceptable simplification by the clinical experts, unlikely to substantially affect the assessment of the therapy’s value.

We used the term diabetes-related complications (DRC) as meaning diabetes-related complications worse than at baseline and additional to hypoglycemia unawareness. Patients from all alive states could get DRC, in which case immunosuppression had to be ended. There was no evidence to support the differential modelling of DRC profiles for IIT patients and transplant patients after graft failure, although the clinical expectation is that the time to complications will be delayed for transplant patients [2, 26–31].

In both treatment arms the same DRC occurred, but patients with graft survival were at lower risk. The risks of initial or major immunosuppressive complications were the same for patients with full and partial graft function. Patients with immunosuppressive complications that were to difficult to treat while on immunosuppression had to end that medication and their graft failed.

### Parameter estimation and implementation

The following subsections describe the probability, cost and utility inputs of our model. We assigned distributions to most parameters (Tables 1, 2 and 3). Further information about the choice of distributions can be found in Appendix 1 (Additional file 1).

**Table 2:** Utilitiy weights and disutility parameters (annual)

Condition	Weight	Disutility (Decrement)	SD	RSD	Distribution	Hyperparameters	Source^a^
Type 1 diabetes (T1DM) no complications^b^	0.81	0.01					[38, 39]
Full graft function no complications^c^	0.82	[Base value]	0.041	5 %	Beta	**α = 70.56**	T1DM no complications plus “no-injection bonus” minus immunosuppression disutility (assumptions)
						**β = 15.48**	
Partial graft function no complications	0.81	0.01 (0.01)	0.002	20 %	Gamma	**α = 25.00**	Same as T1DM but with different uncertainty
						**β = 0.0004**	
Hypoglycemia unawareness (intensive insulin therapy)	0.71	0.11 (0.10)	0.020	20 %	Gamma	**α = 25.00**	Assumption based on [44]
						**β = 0.004**	
*Diabetes-related complications (additional to hypoglycemia unawareness)*							
Amputation	0.60	0.22					Assumption based on [39, 47]
Blindness or severe vision loss	0.63	0.19					Assumption based on [39, 47]
Nephropathy	0.47	0.35					Assumption based on [47]
Heart failure	0.55	0.27					Assumption based on [45]
Stroke	0.49	0.33					Assumptions based on [46]
Myocardial infarction	0.49	0.33					
Angina pectoris	0.62	0.20					
Aggregated diabetes-related complications	0.57	0.25 (0.14)	0.042	30 %	Gamma	**α = 11.11**	Weighted mean of 7 complications above with frequency data from [33] minus 0.03 to account for multiple complication cases
						**β = 0.0126**	
*One-time disutilities (per occurrence)*							
Initial immunosuppressive or other complications	X-0.05	0.05	0.010	20 %	Gamma	**α = 25.00**	Assumption based on [2] and expert opinion
						**β = 0.002**	
Subsequent major immunosuppressive complications	X-0.10	0.10	0.025	25 %	Gamma	**α = 16.00**	Assumption based on [2] and expert opinion
						**β = 0.00625**	

Bold values were directly used as model inputs and for calculating mean, standard deviation (SD) and relative standard deviation (RSD; i.e., SD as percentage of the mean). Other values are rounded and were incorporated into bold values. Superscripts: ^a^ Source relates to mean values. SD values were the authors’ estimations due to lack of data. ^b^ All other conditions also included type 1 diabetes and where applicable hypoglycemia unawareness. ^c^ From this value the corresponding disutility increments of the other conditions were subtracted

**Table 3:** Probability and ratio parameters

Parameter	Mean	SD	RSD	Distribution	Hyperparameters	Source^a^
Ratio of patients having initial complications	0.650	0.0650	10 %	Beta	**α = 34.30**	Assumption based on [2] and expert opinion
					**β = 18.47**	
*Ratio of patients being insulin independent 23 days after the latest transplantation*						
For the 1st transplantation	0.150	0.0375	25 %	Beta	**α = 13.45**	Assumption based on [23] and expert opinion
					**β = 76.21**	
For the 2nd transplantation	0.700	0.1050	15 %	Beta	**α = 12.63**	Assumption based on [23] and expert opinion
					**β = 5.41**	
For the 3rd and 4th transplantation (each)	0.850	0.0355	15 %	Beta	**α = 5.80**	Assumption based on [23] and expert opinion
					**β = 1.02**	
*Hazard ratio (HR) of getting diabetes-related complications*						
For patients with partial graft function	0.450	0.0675	15 %	Log-Normal	**μ = -0.8096**	Assumption based on [2]
					**σ = 0.149166**	
For patients with full graft function	55.56 % of HR with partial graft function (i.e., 0.25)	0.0840	15 %	Log-Normal	**μ = -0.59891**	Assumption based on [2]
					**σ = 0.149166**	
*Recurrence of insulin dependency per cycle*						
Probability of becoming partially dependent for patients with full graft function	0.0077379	0.001161	15 %	Beta	**α = 44.09**	Calculated from [2] and expert opinion
					**β = 5653.02**	
Probability of graft failure for patients with full graft function^b^	0.0000164732	0.00000247	15 %	Beta	**α = 44.44**	Calculated from [2] and expert opinion
					**β = 2,697,667**	
Probability of graft failure within the first six months for patients with partial graft function^b^	0.045	0.00675	15 %	Beta	**α = 42.40**	Assumption based on [23] and expert opinion
					**β = 899.81**	
Probability of graft failure after the first six months for patients with partial graft function^b^	0.00532	0.000798	15 %	Beta	**α = 44.20**	Calculated from [2] and expert opinion
					**β = 8264.00**	
*Complications*						
*Probability of complications per cycle (over model horizon)*						
Major immunosuppressive-related	0.00006201 (0.015)	0.000009302 (0.00225)	15 %	Beta	**α = 44.44**	Assumption based on [2] and expert opinion
					**β = 716,613.00**	
Ratio of above that are have to end immunosuppression	0.100	0.015		Beta	**α = 39.90**	Assumption based on expert opinion
					**β = 359.10**	
Additional diabetes-related^c^	0.0018185 (0.55)	0.000364 (0.0825)	20 %	Beta	**α = 24.96**	Assumption based on [33]
					**β = 13,700.60**	
*Mortality (probability of death)*						
Background all-cause mortality	**(Age-specific)**			Fixed		Weighted mean of [34] fitted to match [35]
Hazard ratio (HR) of mortality due to hypoglycemia	2.40	0.24	10 %	Log-Normal	**μ = 0.870494**	Assumption based on [14, 16, 17, 35] and expert opinion
					**σ = 0.099751**	
HR of mortality due to diabetes-related complications	298.45 % of HR with only hypoglycemia (i.e., 7.16)	29.85	10 %	Log-Normal	**μ = 1.088457**	Assumption based on [14, 16, 17, 35] and expert opinion
					**σ = 0.099751**	

Bold values were directly used as model inputs and for calculating mean, standard deviation (SD) and relative standard deviation (RSD; i.e., SD as percentage of the mean). Other values were incorporated into bold values. Most per-cycle values were used in several states and therefore do not sum up to annual or model horizon values. Superscripts: ^a^ Source relates to mean values. SD values were the authors’ estimations due to lack of data. ^b^ Meaning graft failure that is not due to ending immunosuppression because of major immunosuppressive complications. ^c^ For patients that did not get an islet transplantation or patients with graft failure after islet transplantation

#### Event probabilities

Patient life expectancy is a function of the transition probabilities over the time horizon of the model. Due to data limitations, all probabilities were assumed to be constant over all cycles within each of the simulations, except when directly concerning transplantations or background mortality. While the probabilities for initial transplant success and initial complications could be directly implemented, the majority of probabilities had to be calibrated to match source data (e.g., graft survival) due to ‘competing risk’ bias [32]. The following four sections and Table 4 provide details on the probabilities that were implemented in the model.

**Table 4:** Main results for different scenarios ordered by life expectancy assumptions (means per patient)

Scenario		Cost			Benefit			ICER
Index	Description	Standard care	Islet cell transplantation	Cost difference	Standard care	Islet cell transplantation	Benefit difference	
	*Structural scenarios with difference in LE = 12 years* ^a^							
1	5 % discount rate (base case)	$56,560	$347,377	$290,816	9.59	11.52	1.94	$150,006
2	3.5 % discount rate	$67,363	$369,647	$302,284	11.14	13.51	2.37	$127,278
3	3 % discount rate	$71,695	$378,532	$306,837	11.76	14.31	2.56	$120,008
4	Undiscounted	$109,303	$455,743	$346,440	17.08	21.36	4.28	$80,917
5	5 % discount rate; life years	$56,560	$347,377	$290,816	14.08	15.12	1.05	$278,188
6	Undiscounted; life years	$109,303	$455,743	$346,440	25.30	28.63	3.33	$104,177
7	Undiscounted; double disutilities	$109,303	$455,743	$346,440	14.29	19.86	5.56	$62,254
8	Generic Imm.^b^	$56,560	$273,741	$217,180	9.59	11.52	1.94	$112,023
9	Generic Imm.^b^; 1 % discount rate	$94,062	$320,265	$226,203	14.93	18.48	3.55	$63,668
10	Generic Imm.^b^; each transplantation costs $20,000 less	$56,560	$231,843	$175,283	9.59	11.52	1.94	$90,412
11	Increased costs in IIT (110 %) and DRC (125 %) states	$66,426	$352,527	$286,100	9.59	11.52	1.94	$147,573
12	As row above but undiscounted and doubled disutilities in IIT and DRC states	$129,361	$468,444	$339,083	14.29	19.86	5.56	$60,932
	*Structural scenarios with difference in LE = 10 years* ^a^							
13	5 % discount rate	$60,863	$349,993	$289,130	9.93	11.73	1.80	$160,394
14	3 % discount rate	$78,527	$382,868	$304,341	12.30	14.66	2.36	$128,877
15	Undiscounted; life years	$123,916	$465,610	$341,694	27.20	29.94	2.74	$124,804
	*Structural scenarios with difference in LE = 14 years* ^a^							
16	5 % discount rate	$51,693	$344,471	$292,778	9.22	11.31	2.09	$140,095
17	3 % discount rate	$64,301	$373,931	$309,630	11.20	13.96	2.77	$111,867
18	Undiscounted; life years	$94,621	$446,014	$351,393	23.43	27.36	3.93	$89,402

All scenarios used the base case assumptions with the described structural deviations. Benefit measure is QALY unless noted otherwise. All result numbers are rounded and including sampling variation. Superscripts: ^a^ The assumed difference in life expectancy (LE) caused by hypoglycemia unawareness compared to the non-diabetes population used in the model. ^b^ This scenario assumes generic version immunosuppression

#### Treatment effectiveness

The primary goals of islet cell transplantation are to avoid hypoglycemia and achieve excellent glycemic control [2]. Insulin independence is also a goal, albeit secondary, and it increases the probability of long-lasting graft survival. Another effect of islet cell transplantation is a reduction in DRC [2, 26–31]. We assumed all alive patients without DRC have some graft survival in the cycle after transplantation, because almost all patients become c-peptide positive after the first transplantation. The proportion of patients with full graft function after transplantation, assumed to be increasing from first to third and fourth transplantation, was based on published data [23] and on expert opinion that most patients become insulin independent after the second transplantation. Transition probabilities for graft loss and recurrence of insulin independence were calibrated to achieve the five-year rates (50 % full and 82 % overall graft survival). To simulate the occasional ineffectiveness of the treatment, our model presumed a higher rate of early graft failure over the first six months.

#### Diabetes-related complications

An Ontario study found that 40 % of all diabetes patients get DRC [33]. Our patient population had up to moderate comorbidities and unstable T1DM, a complication in itself which can cause more severe complications including long-term disability through neuroglycopenia [15]. Therefore, we made the assumptions that our patients’ risks of getting other additional DRC are 55 % over the model horizon and that those risks are reduced by 75 % (55 %) when having full (partial) graft function. The risk reductions were implemented through hazard ratios and after graft failure, risks returned to non-transplantation levels.

#### Mortality

We combined age-specific background mortality rates in Alberta from female and male life tables [34], using an age-specific weighted mean (55 % female). Then we modeled differences in life expectancy between our patient population and the general non-diabetic population. A publication reported a difference in life expectancy of 6.5 years between Canadians with and without diabetes at our cohort age (77.7 versus 84.2 years total) [35]. This difference is dominated by the majority of patients who have type 2 diabetes. Although T1DM is considered to have a comparatively higher life-expectancy impact of up to 15 years [14], published data on the magnitude was scarce. Due to the characteristics of our patients, we assumed a 12-year difference for our IIT-treated patients, resulting in a life expectancy of 72.2 years total.

This difference was implemented with two-step increased hazard ratios of mortality in non-graft-survival states, compared to background mortality. For graft survival we took background mortality since there was no evidence of increased mortality due to islet cell transplantation [2]. Of the two data sources for background mortality only one reported diabetes and non-diabetes populations separately. To use both sources, we fitted the all-population life table values from Statistics Canada to make them consistent with the published life expectancy estimate for the non-diabetes population (see Appendix 3 in Additional file 1).

#### Treatment and immunosuppressive complications

Immunosuppressive medication can lead to a range of complications with substantial variation in their severity. Although islet cell transplant can be considered minimally invasive and non-surgical when compared to whole organ transplantation, there are potential complications associated with the transplant procedure itself [2]. We assumed 65 % of the patients experience minor initial complications, which can be overcome within one cycle e.g., by adapting medication [2]. Based on expert opinion we assumed that 1.5 % of patients get major immunosuppressive complications and 10 % of them have to stop immunosuppression as a result. As a simplification, both these complications were implemented into the model as variables of the overall transition probabilities, state costs and utilities.

#### Costs

On the one hand islet cell transplantation causes cost savings from reduced DRC and to a smaller degree insulin therapy [10]. On the other hand it induces additional costs from organ procurement, islet processing, the transplantation procedure itself, follow-up visits, immunosuppression and possibly treatment of complications [2]. We obtained cost data from the University of Alberta hospital (Chris Broscheit via Mike Bentley, personal communication, October 12, 25 and November 6, 2012). The provincial healthcare provider covers costs for procedures, initial drugs and, through a specialized high cost drug program, immunosuppression [36].

Resource use for the graft survival states consisted of: immunosuppression, clinical follow-up, insulin therapy and other medication. (Table 1). We assumed the price of generic immunosuppressant drugs to be a third of the branded price, based on 2014 online market prices of generic versions available in Canada. To account for the need of exogenous insulin in some patients who received beta cell transplants, we added 40 % and 16 % of the IIT costs to the costs in our Partial graft function and Transplantation states correspondingly. The costs of initial and major immunosuppressive complications were assumed to be $600 and $6500 per occurrence respectively. Published cost data on costs for patients with hypoglycemia unawareness, or even T1DM alone, was scarce. We calculated costs in IIT states (with and without DRC) from corrected values (see Appendix 3 in Additional file 1) derived from a study in Ontario [33], which reported 10 year costs of newly diagnosed diabetes patients with and without complications.

#### Utilities

We used utility measures to enable QOL adjustments of life expectancy [37]. The highest utility was assigned to insulin independence. Two studies about health-related QOL with diabetes on type 2 and type 1 and 2 respectively reported a mean utility value of 0.81 for diabetes without complications [38, 39]. We assumed a “no-injection bonus” of 0.02 and a disutility of immunosuppression of 0.01. These led us to 0.82 for insulin independence. All other utility weights were modelled as disutilities subtracted incrementally from this value. Several studies suggested an increased health-related QOL after islet cell transplantation [40–42] or a reduction in fear of hypoglycemia [43]. For partial graft function we assumed a utility of 0.81 because there is some need for insulin injections. In the first cycle we assigned the transplantation state the same utility as IIT. Subsequently it took the same utility value as for partial graft function. We found no published evidence on the utility decrement for severe non-self limiting hypoglycemia. A study of T1DM found disutilities of up to 0.08 for brief and mostly self-limiting hypoglycemia [44]. We assumed a utility decrement of 0.10. We regard this to be a conservative assumption because severe non-self-limiting hypoglycemia events often require third-party assistance.

We used a weighted average of published utility values of seven complication disutilities to calculate the disutility for the DRC state (Table 2) [33, 39, 45–47]. We assumed the following mean one-time disutilities, which were subtracted per occurrence from the state utilities: 0.05 for initial complications and 0.10 per episode for major immunosuppressive complications. When these lasted longer than one cycle, the disutility was applied again.

### General analysis

We conducted a probabilistic analysis and structural sensitivity analysis to investigate the cost-effectiveness of islet cell transplantation and to evaluate uncertainty around our results. The model was constructed and run with the software TreeAge Pro 2015 (Williamstown, MA, USA). Costs and benefits were discounted at 5 % per year, as recommended by the Canadian Agency for Drugs and Technologies in Health [48]. A half-cycle correction was applied.

We performed a probabilistic sensitivity analysis (PSA) to incorporate parameter uncertainty by specifying each parameter as a probability distribution rather than as a single value known with certainty. Monte Carlo simulation was used to propagate the uncertainty regarding the model inputs through to uncertainty regarding the outputs (i.e., expected costs and effects of each intervention). Then the incremental cost effectiveness ratios (ICERs) were calculated using the expected costs and effects of each intervention produced by this probabilistic analysis. In our value of information (VOI) analysis we also calculated the expected value of perfect information (EVPI) and the expected value of perfect parameter information (EVPPI) on both per-patient and population levels. For information on the calculation of the population level values see Appendix 4 (Additional file 1).

The EVPPI analysis used 600 ‘outer’ and 600 ‘inner’ loops. This setting was computationally feasible while showing results very similar to settings with higher numbers of loops (see Appendix 5 in Additional file 1). We also explored using a novel online tool, the Sheffield Accelerated Value of Information (SAVI) application [49], that estimates the EVPPI from given sample outcomes and inputs (parameter draws) of a PSA. We adhered to the traditional calculation approach using nested Monte Carlo simulations, since this allowed us to calculate the EVPPI for various willingness-to-pay levels automatically.

Our EVPPI analysis used three parameter groups: ‘costs’, ‘natural history’, and ‘effectiveness and safety’. Those groups were chosen based on the research vehicle that could be used to collect data informing the parameters. For example, effectiveness and safety data could be collected during a trial that is accompanied by a patient-level QOL study, while cost data could be collected using routine databases or micro-costing studies. For details on each group see Appendix 6 (Additional file 1).

Currently there is no publicly adopted willingness-to-pay threshold i.e., cost-effectiveness threshold for Canada [50]. A study from 2015 assumed $50,000 per QALY gained for CADTH while a threshold of $100,000 was mentioned as early as 1993 [51, 52]. We used both thresholds because we considered $50,000 the more conventional figure and $100,000 the upper limit. Some results at $50,000 were not reported if they are zero or directly follow from our conclusions at $100,000.

### Scenario analysis

We used scenario analyses to consider the impact on the ICERs of different discount rates, unit costs of immunotherapy, mortality rates and disutilities in the IIT states. We examined the impact of assuming (a) 10 year and (b) 14 year reductions in life expectancy. The presumed disutilities of being in the IIT states with and without complications, both of which translate in potential QOL gains through transplantation, could be seen as conservatively small. That is why we looked at the effect of doubling both disutilities. This meant using mean utilities of 0.43 and 0.61 for the IIT states with and without complications respectively. Also we took a preliminary look at some hypothetical scenarios. In one scenario, there was no need for immunosuppressive medication. In another one there was reduction of $20,000 in the cost per transplantation.

Further, we explored the effects of using different costs in the IIT states with and without DRC. This was necessary because (a) the data in our source study from Ontario pointed towards costs that rise with the number of years a patient has diabetes, and (b) our patient group had diabetes for a longer time than the patient group in the source study. We tried to implement the rising costs by using tunnel states for DRC and IIT states, but found this to raise costs slightly and increase model running time more than 40-fold. Therefore, we instead multiplied the drawn values by 1.25 and 1.10 for patients with and without DRC respectively, giving the same result as using tunnel states.

## Results

In our base-case probabilistic analysis, the ICER for islet cell transplantation compared with IIT was $150,006 per QALY gained. This was calculated from expected incremental per-patient cost increases of $290,816 and effects of 1.94 QALYs. We found that transplantations led to prolonging life by on average 3.33 years (undiscounted) per patient and to a substantial increase in quality of life. The latter follows from the incremental figures for QALYs being higher than the corresponding life years figures in Table 4, which shows the main results for different sub model scenarios.

In all simulations and scenarios, islet cell transplantation was more effective but more costly than IIT. At a willingness-to-pay for each additional QALY (WTP) of $50,000 ($100,000) the incremental net monetary benefit of islet cell transplantation was negative (-) $193,881 (-$96,946). Treatment was more costly than IIT even when looking only at costs occurring after the first year. The probability that islet cell transplantation is cost-effective reached 95 % at a WTP of $196,000 and it fell to 13 % at a WTP of $125,000 and 0.5 % at a WTP of $100,000.

**Fig. 2:**
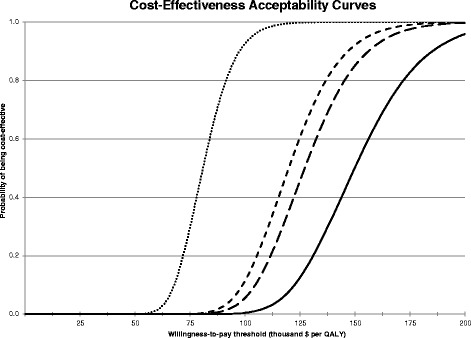
Cost-Effectiveness Acceptability Curves (CEACs). The probability of islet transplantation being cost-effective decreases with increasing discount rate and rises with WTP threshold levels. The CEACs are for scenarios with different discount rates: 0 % (*doted line*), 3 % (*short-dashed line*), 3.5 % (*long-dashed line*) and 5 % (*solid line*). The uncertainty spread around the mean cost-effectiveness increases (*the curves becoming less steep*) with increasing discount rates

**Fig. 3:**
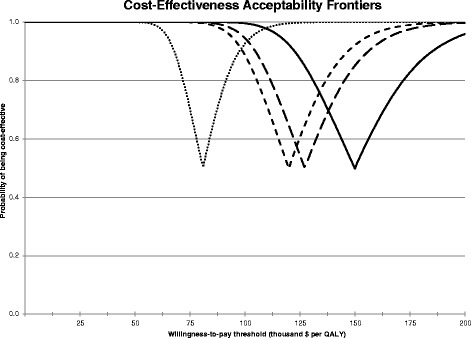
Cost-Effectiveness Acceptability Frontiers (CEAFs). The CEAFs are for scenarios with different discount rates: 0 % (*doted line*), 3 % (*short-dashed line*), 3.5 % (*long-dashed line*) and 5 % (*solid line*). The dents in CEAFs, with their lowest points indicating the willingness-to-pay levels when islet transplantation becomes the net benefit maximizing alternative, move to higher WTP levels when using higher discount rates

Results differed for sub models (Figs. 2 and 3 and Table 4). The lowest ICER for islet cell transplantation ($63,668 per QALY) was found when we assumed generic immunosuppression and a 1 % discount rate. In this scenario the probability of being cost-effective at a WTP of $50,000 or $100,000 was 9.5 % or 99.2 % respectively. The highest ICER ($160,394 per QALY) was found in the scenario that assumed a life expectancy difference of only 10 years between our patients and the non-diabetes population and a 5 % discount rate.

**Fig. 4:**
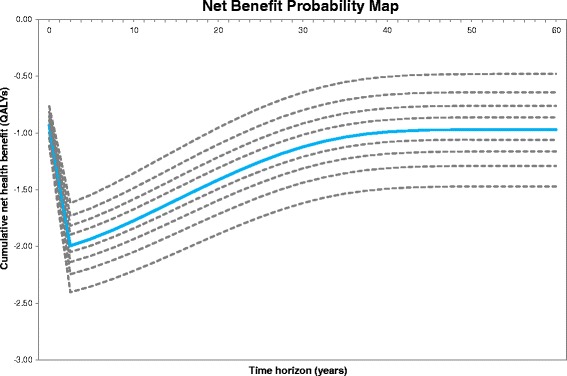
Net Benefit Probability Map (NBPM). Our long-term results showed a higher than 90 % risk that the long-term net health benefit of islet transplantation is lower than minus 0.48 (mean = -0.97). For clarity the data was plotted in 2.5 year intervals

A net benefit probability map (NBPM), which shows uncertainty contours that link the deciles in the distribution of the net health benefit results over time [53], was plotted at a WTP of $100,000 (Fig. 4). On the map one can see that, from a healthcare provider perspective, we found a higher than 90 % risk that in the long-term the net health benefit of transplantation is negative. In addition, the map revealed that events at the beginning of the model horizon dominated the overall variation of the long-term results. The EVPPI results (see below) showed the large initial spread on the NPBM was mostly due to high variation in the cost parameters.

### Scenarios analysis

Compared to the base case, the ICER was substantially and increasingly lowered when using lower discount rates (Table 4). While the difference between 5 and 3 % ICERs was large (~$30,000), neither of these discount rates led to cost-effectiveness at WTP = $100,000. Higher discount rates implicitly favour large effects with a short duration that occur soon over small effects with a long duration, even if the total undiscounted effect is the same. Although treatment benefit of islet cell transplantation is typically spread out over a period of 10 to 15 years, the bulk of the extra costs of transplantation occurs at the start of treatment. With an increasing discount rate, the probability of islet cell transplantation being net-benefit maximising decreased (Fig. 3) and the cost-effectiveness acceptability curves became more flat, which means the range of uncertainty around the expected cost-effectiveness increased (Fig. 2).

Unsurprisingly, a lower disease impact on life expectancy led to islet cell transplantation having lower potential and actual benefits as well as higher costs, and vice versa (Table 4). A lower (higher) impact increased (decreased) the ICER by about $10,000. The scenario with generic immunosuppression and a $20,000 reduction in per-transplantation costs had an ICER of $90,412. Even switching to generic immunosuppression alone lowered incremental costs by $73,636 and the ICER to $112,023. In the scenario with the increased cost in the IIT and DRC states costs of both alternatives were higher but more so in the comparator arm, leading to a slightly lower than base case ICER of $147,573. When looking at doubling the presumed disutilities in the IIT states we found that this increased the cost effectiveness of islet cell transplantation. However, our analysis found that, even together with increased costs in the IIT states and a 0 % discount rate, this was only enough to consider transplantations cost-effective at a WTP of $100,000 but not at a WTP of $50,000 (Table 4).

### Value-of-information analysis

**Fig. 5:**
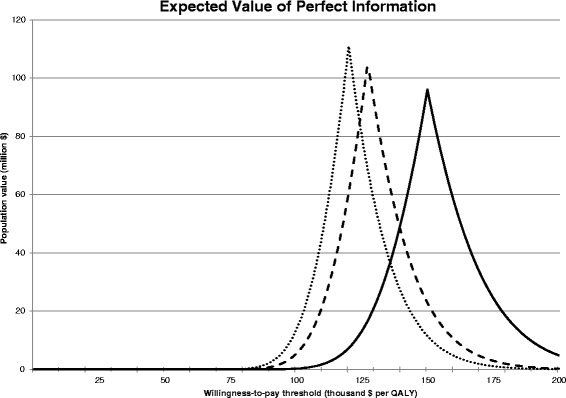
Expected value of perfect information (EVPI). The population EVPI for scenarios with different discount rates depicted as dotted (3 %), dashed (3.5 %) and solid (5 %) lines. With increasing discount rate the uncertainty spread is wider but the EVPI maximum is lower. Population-level factors were adjusted for different discount rates in scenarios

At a WTP of $100,000 the base case EVPI was $50.73 per patient and $260,744 for Alberta, assuming that there are 5140 patients in Alberta who could potentially benefit from the therapy (Fig. 5) [12, 14–17, 54, 55]. Using a 3 % discount rate the EVPI was $2285 per patient and $13,44 million for Alberta. With generic immunosuppression those figures increased to $6480 and $33.31 million. However, the EVPI was zero at a more conventional WTP of $50,000. This result was sensitive to the choice of discount rate. When we used a discount rate of 1 % and generic immunosuppression the EVPI at a WTP of $50,000 was $1493 per patient and $14.34 million for Alberta. Depending on the WTP threshold and discount rate, this means that further research into whether islet cell transplantation is cost-effective can be worthwhile for Alberta up to this upper limit. The value to Canada of investing further research around islet cell transplantation would be substantially greater, as Alberta is home to a minority of Canadian people with unstable Type 1 Diabetes.

To compare the results for the parameters groups of our EVPPI analysis we report the per-patient maximum value for each group of parameters, that is the value at a WTP equal to the respective ICER. That value was $12,267 for the costs group, $11,401 for the natural history group and $8073 for the effectiveness and safety group.

## Discussion

We found that islet cell transplantation is not cost-effective when compared to standard therapy for unstable T1DM. Although it shows large improvements in health outcomes over standard therapy, as anticipated it is also much more costly. These extra costs are not offset by more than proportionate improvements in health outcomes. There is low value in obtaining further information to reduce uncertainty in the model parameters. Those results were found to be robust across a wide range of alternative assumptions. However, the use of generic immunosuppressive medication alone could greatly improve cost-effectiveness.

Our study compared several structurally different scenarios giving insight into the role of key parameters in the model. For example, we showed that one particular model input, although independent of the treatments under consideration, had a substantial impact on the ICER. The discount rate was important in estimating the net benefit of our treatment strategies. Future studies may wish to consider including it as a ‘parameter of interest’ for their VOI analysis.

The value of further research into the cost-effectiveness of islet cell transplantation is dependent on lowering treatment costs, the choice of discount rate and the willingness to pay for health. Resolving the uncertainty around cost parameters would most valuable. One purpose of our scenarios was to investigate the VOI implications of future cost changes. While this study estimated the VOI only for Alberta, the value of the public information generated from further research there is likely to have value in informing decision making in other jurisdictions. Hence the value to Canada will be substantially higher and the value to the international diabetes community greater still. If the willingness to pay for health is substantially higher than used in our analysis, such as the $150,000 to $200,000 suggested by Neumann and colleagues for the United States, then further research would be extremely valuable and routine adoption would be a more cost-effective use of limited healthcare resources [56].

We found two other cost-effectiveness studies that looked at allogeneic islet cell transplantation alone (i.e., without other transplantations) and both had IIT as comparator. Both studies and our results show that islet cell transplantation provides important improvements in health outcomes. The first study adopted various different assumptions from our study [57]. For example, the model assumed a 20 year time horizon and assumed that transplantation reduced the risk of DRC to zero. The study also assumed a much lower age at the start of treatment (20 years), which is substantially lower than observed in practice. Whilst the study found that transplant could be cost saving to the system, it was driven by graft survival assumptions which the authors acknowledged to be optimistic. In addition, the distributions used to characterise parameter uncertainty did not adhere with best practice [20].

The second study was conducted by the Alberta-based Institute of Health Economics (IHE) and had a series of different model scenarios [23]. The scenario closest to our base case showed overall results similar to those indicated by our study. That lifetime sub model calculated an ICER of $163,387 (in 2012 prices) if graft survival completely prevented DRC. Whilst there were differences in the cohort demographic characteristics and the costs of transplantation, the most important difference between our study and the IHE study was the assumption regarding the impact of transplantation on the risk of diabetes-related complications. The IHE analyses assumed in the compared scenario a much greater risk reduction (and in other scenarios no risk reduction). However, the impact of assuming complete prevention of DRC on the ICER was largely offset by higher transplantation costs and lower treatment costs for IIT.

A limitation of this study is that IIT and islet cell transplantation currently cannot be considered mutually exclusive alternate or equivalent therapies. Islet cell transplantation is reserved as an end-stage therapy for subjects where IIT has been offered, optimized and has still failed to resolve risk of incapacitating and potentially life-threatening recurrent hypoglycemic events. Nevertheless, for lack of alternatives besides transplantation, IIT is still standard therapy for these individuals. For those patients for which IIT has completely failed, one can argue - aside of cost-effectiveness considerations - that provision of islet cell transplantation is still an appropriate use of healthcare resources, because here all the criteria of the ‘rule of rescue’ apply [58].

Unstable T1DM has a relatively low prevalence of about 0.1 % of the population and islet cell transplantation is still a comparatively new technology. Therefore only a limited amount of published data was available to be used as input for model parameters. One should take this into consideration when interpreting our results. Additionally we based the costs for non-graft-survival states on values from Ontario rather than Alberta. However, relevant coverage policies were found to be similar in both provinces.

Parameter correlation was implemented only to a limited extent because of a lack of adequate data. This is a clear limitation of our study. State costs were composite variables composed of relevant cost draws. The reduced risk of DRC and the higher risk of death without graft function were each modelled with nested two-step hazard ratios. While limited parameter correlation may have influenced our findings, it could be argued that parameter correlations are not as important in cohort models as they are in individual patient simulations, thereby limiting any impact.

## Conclusions

Islet cell transplantation, although highly effective, is currently not cost-effective due to its increased costs. Although there are uncertainties in many model inputs, the value of obtaining further information to inform a cost-effectiveness analysis is low when currently recommended discount rates are applied. If further research were to be conducted, research about procedure and medication-related costs would have the highest research value because of the relatively large uncertainty around those parameters and their importance in determining the current results. Further, we suggest the value of information should not only be derived from current data alone when knowing that this data will most likely change in the future.

### Ethics approval

This study meets the ethical standards implemented by the Research Ethics Office of the University of Alberta, who approved the research proposals connected with this minimum-risk non-clinical study.

### Availability of data and materials

The datasets supporting the conclusions of this article are available in the Open Science Framework repository, DOI 10.17605/OSF.IO/5QJPV. Further we also include here an additional file (Additional file 1.docx).

The additional file contains the appendix which has seven short sections that complement the main manuscript by providing details deemed to technical for inclusion in the main manuscript. The section headings are: Appendix 1: Parameter distributions, Appendix 2: Usage of corrected cost data, Appendix 3: Normalising background mortality rates, Appendix 4: Population-level EVPI factor, Appendix 5: EVPPI sample and simulation loops, Appendix 6: Value of information parameter groups, and References in the Appendix.

## Additional file

Additional file 1: Appendix 1: Parameter distributions. Appendix 2: Usage of corrected cost data. Appendix 3: Normalising background mortality rates. Appendix 4: Population-level EVPI factor. Appendix 5: EVPPI sample and simulation loops. Appendix 6: Value of information parameter groups. (DOCX 29 kb)

## Abbreviations

DRC: diabetes-related complications
EVPI: expected value of perfect information
EVPPI: expected value of perfect parameter information
ICER: incremental cost-effectiveness ratio
IIT: intensive insulin therapy
PSA: probabilistic sensitivity analysis
QALY: quality-adjusted life-year
QOL: quality of life
RSD: relative standard deviation
SD: standard deviation
T1DM: type 1 diabetes melitus
VOI: value of information (decision analytic context)
WTP: willingness to pay per additional QALY

## References

[1] Ryan EA, Shandro T, Green K, Paty BW, Senior PA, Bigam D, et al. Assessment of the severity of hypoglycemia and glycemic lability in type 1 diabetic subjects undergoing islet transplantation. Diabetes. 2004; doi:10.2337/diabetes.53.4.955. 10.2337/diabetes.53.4.955 15047610

[2] Senior PA, Kin T, Shapiro J, Koh A. Islet transplantation at the University of Alberta: Status update and review of progress over the last decade. Can J Diabetes. 2012; doi:10.1016/j.jcjd.2012.01.002. Elsevier Ltd.

[3] Jamiolkowski RM, Guo LY, Li YR, Shaffer SM, Naji A (2012). Islet transplantation in type I diabetes mellitus. Yale J Biol Med.

[4] Shapiro J, Lakey JRT, Ryan EA, Korbutt GS, Toth E, Warnock GL, et al. Islet transplantation in seven patients with type 1 diabetes mellitus using a glucocorticoid-free immunosuppressive regimen. N Engl J Med. 2000; doi:10.1056/NEJM200007273430401. 10.1056/NEJM200007273430401 10911004

[5] Steele C, Hagopian WA, Gitelman S, Masharani U, Cavaghan M, Rother KI (2004). Insulin secretion in type 1 diabetes. Diabetes.

[6] Hanas R. Dead-in-bed syndrome in diabetes mellitus and hypoglycemic unawareness. Lancet. 1997; doi:10.1016/S0140-6736(05)63081-4. 10.1016/S0140-6736(05)63081-4 9274591

[7] Cryer PE. mechanisms of hypoglycemia-associated autonomic failure and its component syndromes in diabetes. Diabetes. 2005; doi:10.2337/diabetes.54.12.3592. 10.2337/diabetes.54.12.3592 16306382

[8] Barton FB, Rickels MR, Alejandro R, Hering BJ, Wease S, Naziruddin B, et al. Improvement in outcomes of clinical islet transplantation: 1999-2010. Diabetes Care. 2012; doi:10.2337/dc12-0063. 10.2337/dc12-0063 PMC3379615 22723582

[9] Ryan EA, Paty BW, Senior PA, Bigam D, Alfadhli E, Kneteman NM (2005). Five-year follow-up after clinical islet transplantation. Diabetes.

[10] Collaborative Islet Transplant Registry (CITR). CITR - Seventh Annual Report (2010). Rockville, Maryland: Collaborative Islet Transplant Registry; 2011.

[11] Shapiro JAM, Toso C, Imes S, Koh A, Kin T, O’Gorman D, et al. Five-year results of islet-alone transplantation match pancreas-alone transplantation with alemtuzumab, Tac/MMF, with strong suppression of auto and alloreactivity. Presentation at the 13th Congress of the International Pancreas and Islet Transplant Association, Prague. Available from: http://www.tts.org/index.php?option=com_tts&view=presentation&id=8004. [Accessed 18 Dec 2013].

[12] Daneman D. Type 1 diabetes. Lancet. 2006; doi:10.1016/S0140-6736(06)68341-4.

[13] Public Health Agency of Canada. Diabetes in Canada: Facts and figures from a public health perspective - Public Health Agency of Canada. Public Health Agency of Canada. Available from: http://www.phac-aspc.gc.ca/cd-mc/publications/diabetes-diabete/facts-figures-faits-chiffres-2011/chap1-eng.php#DIA/. [Accessed 18 Dec 2013].

[14] Canadian Diabetes Association. The prevalence and costs of diabetes. Canadian Diabetes Association. Available from: http://www.diabetes.ca/documents/about-diabetes/PrevalanceandCost_09.pdf. [Accessed 17 Dec 2013].

[15] Merani S, Shapiro JAM. Current status of pancreatic islet transplantation. Clin Sci (Lond). 2006; doi:10.1042/CS20050342. 10.1042/CS20050342 16689680

[16] Cryer PE. The barrier of hypoglycemia in diabetes. Diabetes. 2008; doi:10.2337/db08-1084. 10.2337/db08-1084 PMC2584119 19033403

[17] Skrivarhaug T, Bangstad H-J, Stene L. Long-term mortality in a nationwide cohort of childhood-onset type 1 diabetic patients in Norway. Diabetologia. 2006; doi:10.1007/s00125-005-0082-6. 10.1007/s00125-005-0082-6 16365724

[18] Chhabra P, Brayman KL. Overcoming Barriers in Clinical Islet Transplantation: Current Limitations and Future Prospects. Curr Probl Surg. 2014; doi:10.1067/j.cpsurg.2013.10.002. 10.1067/j.cpsurg.2013.10.002 24411187

[19] Roberts M, Russell LB, Paltiel AD, Chambers M, McEwan P, Krahn M. Conceptualizing a model: a report of the ISPOR-SMDM Modeling Good Research Practices Task Force-2. Value Health. 2012; doi:10.1016/j.jval.2012.06.016. 10.1016/j.jval.2012.06.016 PMC4207095 22999129

[20] Briggs AH, Weinstein MC, Fenwick EAL, Karnon J, Sculpher MJ, Paltiel AD. Model parameter estimation and uncertainty: a report of the ISPOR-SMDM Modeling Good Research Practices Task Force-6. Value Health. 2012; doi:10.1016/j.jval.2012.04.014. 10.1016/j.jval.2012.04.014 22999133

[21] Siebert U, Alagoz O, Bayoumi AM, Jahn B, Owens DK, Cohen DJ, et al. State-transition modeling: a report of the ISPOR-SMDM Modeling Good Research Practices Task Force-3. Value Health. 2012; doi:10.1016/j.jval.2012.06.014. 10.1016/j.jval.2012.06.014 22999130

[22] Hall PS, Hulme C, McCabe CJ, Oluboyede Y, Round J, Cameron DA (2011). Updated cost-effectiveness analysis of trastuzumab for early breast cancer: a UK perspective considering duration of benefit, long-term toxicity and pattern of recurrence. Pharmacoeconomics.

[23] Institute of Health Economics (2013). Islet transplantation for the treatment of type 1 diabetes.

[24] Statistics Canada. CANSIM - 326-0021 - Consumer Price Index (CPI), 2009 basket (extended table version from 2004 onwards). Statistics Canada, CANSIM, table 326-0021. Available from: http://www5.statcan.gc.ca/cansim/a26?lang=eng&retrLang=eng&id=3260021&pattern=&csid=. [Accessed 19 Dec 2013].

[25] Koh A, Imes S, Kin T, Dinyari P, Malcolm A, Toso C, et al. Supplemental islet infusions restore insulin independence after graft dysfunction in islet transplant recipients. Transplantation. 2010; doi:10.1097/TP.0b013e3181bcdbe8. 10.1097/TP.0b013e3181bcdbe8 20145529

[26] Lee TC, Barshes NR, O’Mahony CA, Nguyen L, Brunicardi FC, Ricordi C, et al. The effect of pancreatic islet transplantation on progression of diabetic retinopathy and neuropathy. Transplant Proc. 2005; doi:10.1016/j.transproceed.2005.03.011. 10.1016/j.transproceed.2005.03.011 15964394

[27] Venturini M, Fiorina P, Maffi P, Losio C, Vergani A, Secchi A, et al. Early increase of retinal arterial and venous blood flow velocities at color Doppler imaging in brittle type 1 diabetes after islet transplant alone. Transplantation. 2006; doi:10.1097/01.tp.0000208631.63235.6a. 10.1097/01.tp.0000208631.63235.6a 16699454

[28] Fiorina P, Shapiro J, Ricordi C, Secchi A. The clinical impact of islet transplantation. Am J Transplant. 2008; doi:10.1111/j.1600-6143.2008.02353.x. 10.1111/j.1600-6143.2008.02353.x 18828765

[29] Warnock GL, Thompson DM, Meloche RM, Shapiro RJ, Ao Z, Keown P, et al. A multi-year analysis of islet transplantation compared with intensive medical therapy on progression of complications in type 1 diabetes. Transplantation. 2008; doi:10.1097/TP.0b013e318190b052. 10.1097/TP.0b013e318190b052 19104418

[30] Thompson DM, Begg IS, Harris C, Ao Z, Fung MA, Meloche RM, et al. Reduced progression of diabetic retinopathy after islet cell transplantation compared with intensive medical therapy. Transplantation. 2008; doi:10.1097/TP.0b013e318172ca07. 10.1097/TP.0b013e318172ca07 18497678

[31] Thompson DM, Meloche M, Ao Z, Paty B, Keown P, Shapiro RJ, et al. Reduced progression of diabetic microvascular complications with islet cell transplantation compared with intensive medical therapy. Transplantation. 2011; doi:10.1097/TP.0b013e31820437f3. 10.1097/TP.0b013e31820437f3 21258272

[32] Gooley TA, Leisenring W, Crowley J, Storer BE (1999). Estimation of failure probabilities in the presence of competing risks: new representations of old estimators. Stat Med.

[33] O’Reilly D, Hopkins R, Blackhouse G, Clarke P, Hux J, Guan J (2006). Development of an Ontario diabetes economic model (ODEM) and application to a multidisciplinary primary care diabetes management program. (Report prepared for the Ontario Ministry of Health and Long-term Care).

[34] Statistics Canada. Life Tables, Canada, Provinces and Territories - 2009 to 2011. Statistics Canada, Publication 84-537-X. Available from: http://www.statcan.gc.ca/pub/84-537-x/2013005/tbl-eng.htm. [Accessed 19 Dec 2013].

[35] Public Health Agency of Canada. Figure 2-7. Life expectancy (LE) and health-adjusted life expectancy (HALE) among individuals from birth and older, by age group, sex, and diabetes status, Canada, 2004/05 to 2006/07. Diabetes in Canada: Facts and figures from a public health perspective - Public Health Agency of Canada - Chapter 2 – The health impact of diabetes on Canadians. Available from: http://www.phac-aspc.gc.ca/cd-mc/publications/diabetes-diabete/facts-figures-faits-chiffres-2011/chap2-eng.php#MOR. [Accessed 18 Dec 2013].

[36] Government of Alberta - Ministry of Health. Specialized High Cost Drug Program – Alberta Health. Ministry of Health website. Goverment of Alberta,. Available from: http://www.health.alberta.ca/services/drugs-high-cost.html. [Accessed 17 Dec 2013].

[37] Briggs A, Sculpher M, Claxton K (2006). Decision modelling for health economic evaluation. Handbooks in health economic evaluation. 1st ed.

[38] Currie CJ, Poole CD, Woehl A, Morgan CL, Cawley S, Rousculp MD, et al. The health-related utility and health-related quality of life of hospital-treated subjects with type 1 or type 2 diabetes with particular reference to differing severity of peripheral neuropathy. Diabetologia. 2006; doi:10.1007/s00125-006-0380-7. 10.1007/s00125-006-0380-7 16944094

[39] Clarke P, Gray A, Holman R. Estimating Utility Values for Health States of Type 2 Diabetic Patients Using the EQ-5D (UKPDS 62). Med Decis Mak. 2002; doi:10.1177/0272989X0202200412. 10.1177/0272989X0202200412 12150599

[40] Poggioli R, Faradji RN, Ponte G, Betancourt A, Messinger S, Baidal DA, et al. Quality of life after islet transplantation. Am J Transplant. 2006; doi:10.1111/j.1600-6143.2005.01174.x. 10.1111/j.1600-6143.2005.01174.x 16426323

[41] Tharavanij T, Betancourt A, Messinger S, Cure P, Leitao CB, Baidal DA, et al. Improved long-term health-related quality of life after islet transplantation. Transplantation. 2008;86:1161–7. 10.1097/TP.0b013e31818a7f45 PMC2741424 19005394

[42] Benhamou PY, Milliat-Guittard L, Wojtusciszyn A, Kessler L, Toso C, Baertschiger R, et al. Quality of life after islet transplantation: data from the GRAGIL 1 and 2 trials. Diabet Med. 2009; doi:10.1111/j.1464-5491.2009.02731.x. 10.1111/j.1464-5491.2009.02731.x 19538237

[43] Toso C, Shapiro JAM, Bowker S, Dinyari P, Paty B, Ryan EA, et al. Quality of life after islet transplant: impact of the number of islet infusions and metabolic outcome. Transplantation. 2007; doi:10.1097/01.tp.0000280550.01028.89. 10.1097/01.tp.0000280550.01028.89 17876283

[44] Tabaei B, Shill-Novak J, Brandle M (2004). Glycemia and the quality of well-being in patients with diabetes. Qual Life Res.

[45] Kirsch J, McGuire A (2000). Establishing health state valuations for disease specific states: an example from heart disease. Health Econ.

[46] Tengs TO, Wallace A (2000). One thousand health-related quality-of-life estimates. Med Care.

[47] Palmer AJ, Roze S, Valentine WJ, Minshall ME, Foos V, Lurati FM, et al. The CORE Diabetes Model: Projecting long-term clinical outcomes, costs and cost-effectiveness of interventions in diabetes mellitus (types 1 and 2) to support clinical and reimbursement decision-making. Curr Med Res Opin. 2004; doi:10.1185/030079904X1980. 10.1185/030079904X1980 15324513

[48] Canadian Agency for Drugs and Technologies in Health (CADTH) (2006). Guidelines for the economic evaluation of health technologies: Canada.

[49] Strong M, Breeze P, Thomas C, Brennan A. SAVI - Sheffield Accelerated Value of Information. Available from: http://savi.shef.ac.uk/SAVI/. [Accessed 15 June 2015].

[50] Rocchi A, Menon D, Verma S, Miller E. The role of economic evidence in canadian oncology reimbursement decision-making: to Lambda and beyond. Value Heal. 2008; doi:10.1111/j.1524-4733.2007.00298.x. International Society for Pharmacoeconomics and Outcomes Research (ISPOR). 10.1111/j.1524-4733.2007.00298.x 18179658

[51] Griffiths EA, Hendrich JK, Stoddart SD, Walsh SC. Acceptance of health technology assessment submissions with incremental cost-effectiveness ratios above the cost-effectiveness threshold. Clinicoecon Outcomes Res. 2015; doi:10.2147/CEOR.S87462. 10.2147/CEOR.S87462 PMC4564087 26366099

[52] Laupacis A, Feeny D, Detsky AS, Tugwell PX (1992). How attractive does a new technology have to be to warrant adoption and utilization? Tentative guidelines for using clinical and economic evaluations. CMAJ.

[53] McCabe C, Edlin R, Hall P. Navigating time and uncertainty in health technology appraisal: would a map help? Pharmacoeconomics. 2013; doi:10.1007/s40273-013-0077-y. 10.1007/s40273-013-0077-y 23877738

[54] Philips Z, Claxton K, Palmer S. The half-life of truth: what are appropriate time horizons for research decisions? Med Decis Mak. 2008; doi:10.1177/0272989X07312724. 10.1177/0272989X07312724 18448701

[55] Statistics Canada. Population by year, by province and territory (Number). Statistics Canada, CANSIM, table 051-0001. Available from: http://www.statcan.gc.ca/tables-tableaux/sum-som/l01/cst01/demo02a-eng.htm. [Accessed 17 Jan 2014].

[56] Neumann PJ, Cohen JT, Weinstein MC. Updating cost-effectiveness--the curious resilience of the $50,000-per-QALY threshold. N Engl J Med. 2014; doi: 10.1056/NEJMp1405158. 10.1056/NEJMp1405158 25162885

[57] Beckwith J, Nyman JA, Flanagan B, Schrover R, Schuurman H-J. A health economic analysis of clinical islet transplantation. Clin Transplant. 2012; doi:10.1111/j.1399-0012.2011.01411.x. 10.1111/j.1399-0012.2011.01411.x 21323736

[58] Cookson R, Mccabe C, Tsuchiya A. Public healthcare resource allocation and the Rule of Rescue. J Med Ethics. 2008; doi:10.1136/jme.2007.021790. 10.1136/jme.2007.021790 18591290

