# Supplementary material for: Cost effectiveness and value of information analyses of islet cell transplantation in the management of ‘unstable’ type 1 diabetes mellitus
Source: BMC Endocr Disord. 2016 Apr 9;16:17. doi: 10.1186/s12902-016-0097-7 (PMC4826503; doi:10.1186/s12902-016-0097-7)
Supplement: Additional file 1: — Appendix 1: Parameter distributions. Appendix 2: Usage of corrected cost data. Appendix 3: Normalising background mortality rates. Appendix 4: Population-level EVPI factor. Appendix 5: EVPPI sample and simulation loops. Appendix 6: Value of information parameter groups. (DOCX 29 kb) [file 12902_2016_97_MOESM1_ESM.docx]

# Appendix 1: Parameter distributions

Here we present an overview of why we chose a particular type of distribution for a specific parameter. We tried to adhere to the relevant best practice guidelines by the Good Research Practices in Modeling Task Force [1]. When more than one type of distribution could be used we looked at which one would better fit the expected uncertainty of that parameter.

For cost parameters we chose log-normal distributions except for complication costs where we used gamma distributions. For utility parameters we chose gamma distributions and one beta distribution. The highest utility state (insulin independence) was assigned a beta distribution considering that its mean was relatively close to one. For the other states we used this beta distribution draw and subtracted disutility decrements that were assigned gamma distributions. This approach was chosen to maintain a logical order between the utilities of the different states according to health conditions. For one-time disutilities we chose gamma distributions as well and took their negative value because they need to be negative and otherwise unbounded.

We assigned log-normal and beta distributions to ratios and transition probabilities. For hazard ratios we applied log-normal distributions as recommended in the guidelines by Briggs and colleagues [1]. To all transition probability parameters, as well as to the ratio of all patients that have initial complications, we assigned beta distributions since those parameters are bounded between 0 and 1.

# Appendix 2: Usage of corrected cost data

An Ontario report from 2006 stated that “on average, complications resulted in an additional $2,061 per patient per year” (page 26) [2]. But other information in report (e.g. Table 4) clearly identify this number to be the difference between the whole group of newly diagnosed diabetes people, with and without complications, and its subgroup of patients without complications. We calculated a corrected difference, using their patient numbers and total costs in Table 4, and used it as model input.

# Appendix 3: Normalising background mortality rates

Statistics Canada’s life table values are derived from the general population, including diabetes patients [3]. This is why when calibrating the model, we saw that applying the newest life expectancy data in Statistics Canada’s life tables for Alberta resulted in a different all-population life expectancy than reported by the Public Health Agency of Canada. That agency’s latest data provided overall Canadian life expectancy figures separated into groups by diabetes status and age but it did not include life tables [4]. Therefore we used a fixed adjustment factor of 0.8942 (derived through model re-calibration to fit a *non*-diabetes population) on top of the Alberta life table values from Statistics Canada in order to adjust the model to the life expectancy figures from the Public Health Agency of Canada.

The initial model calibration was done by first converting annual life table values into per-cycle values and then truncating the model to a two state model (with the states non-diabetes alive and dead) to test if the calculated values lead to the same non-diabetes life expectancy as reported by the Public Health Agency of Canada. We re-calibrated the model using an adjustment factor until the model was accurately producing the life expectancy reported by the Public Health Agency of Canada.

# Appendix 4: Population-level EVPI factor

Following Philips et al. [5], we assumed that the current patient population and all future patients with unstable T1DM could potentially benefit from research into the islet cell transplantation technology, therefore having no limit on the time horizon of the technology. Assuming 0.1% of the whole population had unstable type 1 diabetes [6–10], we calculated that in Alberta the current population which could potentially benefit is 4,025 [11]. Assuming an average life expectancy with unstable diabetes of 72.2 years and everything else being equal, we calculated the number of new cases of unstable type 1 diabetes in Alberta as 56 per year. Using this procedure and the currently recommended 5% discount rate, we calculated a population-level EVPI factor of 5,140 for Alberta (population level factors were adjusted for different discount rates in scenarios). Multiplying this factor by the EVPI for a single patient yields the population-level EVPI for Alberta. The assumption that the number of new cases remains constant may be conservative, implying that these factors may be understated.

# Appendix 5: EVPPI sample and simulation loops

The EVPPI calculation using nested Monte Carlo simulations is computationally intensive and results in a trade-off between feasibility and expected accuracy of EVPPI estimates. Additional loops result in longer simulation durations but more accurate estimates, especially at WTP levels far away from the ICER.

We compared the accuracy of different loop ‘settings’ before running the EVPPI calculations for all relevant scenarios and VOI groups. As a test case, we compared the EVPPI results for the parameter group ‘costs’ in the base case using different inner and outer loop settings, and the same random number seed. The final setting used 600 outer loops and 600 inner loops, which produced positive results at 120 WTP levels (each spaced $1000 apart).

While settings with lower loop numbers were deemed insufficient, the final setting had similar results to those found with double the number of inner loops and the same number of outer loops. For example, the setting with 600 inner and 1200 outer loops had positive results at 119 WTP levels. In 24% of those levels the results of the final setting were within 10% of the more accurate results (see Table A1). The estimates of the maximum VOI, where the WTP is equal to the ICER, were within 2.6% of the other under each approach. In general, the accuracy decreased with distance from the ICER, except at the far outliers where the VOI was zero in any case.

**Table A1:** Accuracy of the final setting compared to the 600x1200 setting. Proportions of positive results with the final setting (600x600) that were within ranges of the results with the 600x1200 setting.

|  | Number of results within the range | Percentage of results within the range |
| --- | --- | --- |
| Within a +-10% range | 29/119 | 24% |
| Within a +-25% range | 59/119 | 50% |
| Within a +-50% range | 98/119 | 82% |

# Appendix 6: Value of information parameter groups

## Cost group

1. All costs occurring anywhere in the model

## Natural history group

1. Probability of getting additional DRC
2. Hazard ratio of mortality due to hypoglycemia (IIT state) compared to background mortality
3. Hazard ratio of mortality due to DRC (DRC state) compared to IIT state
4. Utility in IIT state
5. Utility in DRC state

## Effectiveness and safety group

1. Utility in insulin independent state
2. Utility in partial graft survival state
3. Disutility of initial immunosuppressive or other complications
4. Disutility of major immunosuppressive complications
5. Ratio of patients with initial post-transplant complications
6. Ratios of patients being insulin independent 23 days after the latest transplantation
7. Ratio of patients with major immunosuppressive complications that have to end immunosuppression because of the former (i.e. leading to graft failure).
8. Hazard ratio of getting DRC in the partial graft survival state compared to the IIT state
9. Hazard ratio of getting DRC in the insulin independent state compared to the partial graft survival state

Probabilities of …

1. patients on immunosuppression getting major immunosuppressive complications
2. patients with full graft function becoming partially insulin dependent (partial graft function)
3. patients with full graft function having graft failure
4. patients with partial graft function (within the first 6 months) having graft failure
5. patients with partial graft function (after the first 6 months) having graft failure

# References in the Appendix

[1] Briggs AH, Weinstein MC, Fenwick EAL, Karnon J, Sculpher MJ, Paltiel AD. Model parameter estimation and uncertainty: a report of the ISPOR-SMDM Modeling Good Research Practices Task Force-6. Value Health. 2012; doi:10.1016/j.jval.2012.04.014.

[2] O’Reilly D, Hopkins R, Blackhouse G, Clarke P, Hux J, Guan J, et al. Development of an Ontario diabetes economic model (ODEM) and application to a multidisciplinary primary care diabetes management program. (Report prepared for the Ontario Ministry of Health and Long-term Care). Program for Assessment of Technology in Health (PATH). Hamilton, Ontario, 2006.

[3] Statistics Canada. Life Tables, Canada, Provinces and Territories - 2009 to 2011. Statistics Canada, Publication 84-537-X. Available from: http://www.statcan.gc.ca/pub/84-537-x/2013005/tbl-eng.htm. [Accessed December 19, 2013].

[4] Public Health Agency of Canada. Figure 2-7. Life expectancy (LE) and health-adjusted life expectancy (HALE) among individuals from birth and older, by age group, sex, and diabetes status, Canada, 2004/05 to 2006/07. Diabetes in Canada: Facts and figures from a public health perspective - Public Health Agency of Canada - Chapter 2 – The health impact of diabetes on Canadians. Available from: http://www.phac-aspc.gc.ca/cd-mc/publications/diabetes-diabete/facts-figures-faits-chiffres-2011/chap2-eng.php#MOR. [Accessed December 18, 2013].

[5] Philips Z, Claxton K, Palmer S. The half-life of truth: what are appropriate time horizons for research decisions? Med Decis Mak. 2008; doi:10.1177/0272989X07312724.

[6] Daneman D. Type 1 diabetes. Lancet. 2006; doi:10.1016/S0140-6736(06)68341-4.

[7] Canadian Diabetes Association. The prevalence and costs of diabetes. Canadian Diabetes Association. Available from: http://www.diabetes.ca/documents/about-diabetes/PrevalanceandCost_09.pdf. [Accessed December 17, 2013].

[8] Merani S, Shapiro JAM. Current status of pancreatic islet transplantation. Clin Sci (Lond). 2006; doi:10.1042/CS20050342.

[9] Cryer PE. The barrier of hypoglycemia in diabetes. Diabetes. 2008; doi:10.2337/db08-1084.

[10] Skrivarhaug T, Bangstad H-J, Stene L. Long-term mortality in a nationwide cohort of childhood-onset type 1 diabetic patients in Norway. Diabetologia. 2006; doi:10.1007/s00125-005-0082-6.

[11] Statistics Canada. Population by year, by province and territory (Number). Statistics Canada, CANSIM, table 051-0001. Available from: http://www.statcan.gc.ca/tables-tableaux/sum-som/l01/cst01/demo02a-eng.htm. [Accessed January 17, 2014].
